# Supplementary figures and images for: CYB561 supports the neuroendocrine phenotype in castration-resistant prostate cancer
Source: PLoS One. 2024 May 13;19(5):e0300413. doi: 10.1371/journal.pone.0300413 (PMC11090301; doi:10.1371/journal.pone.0300413)

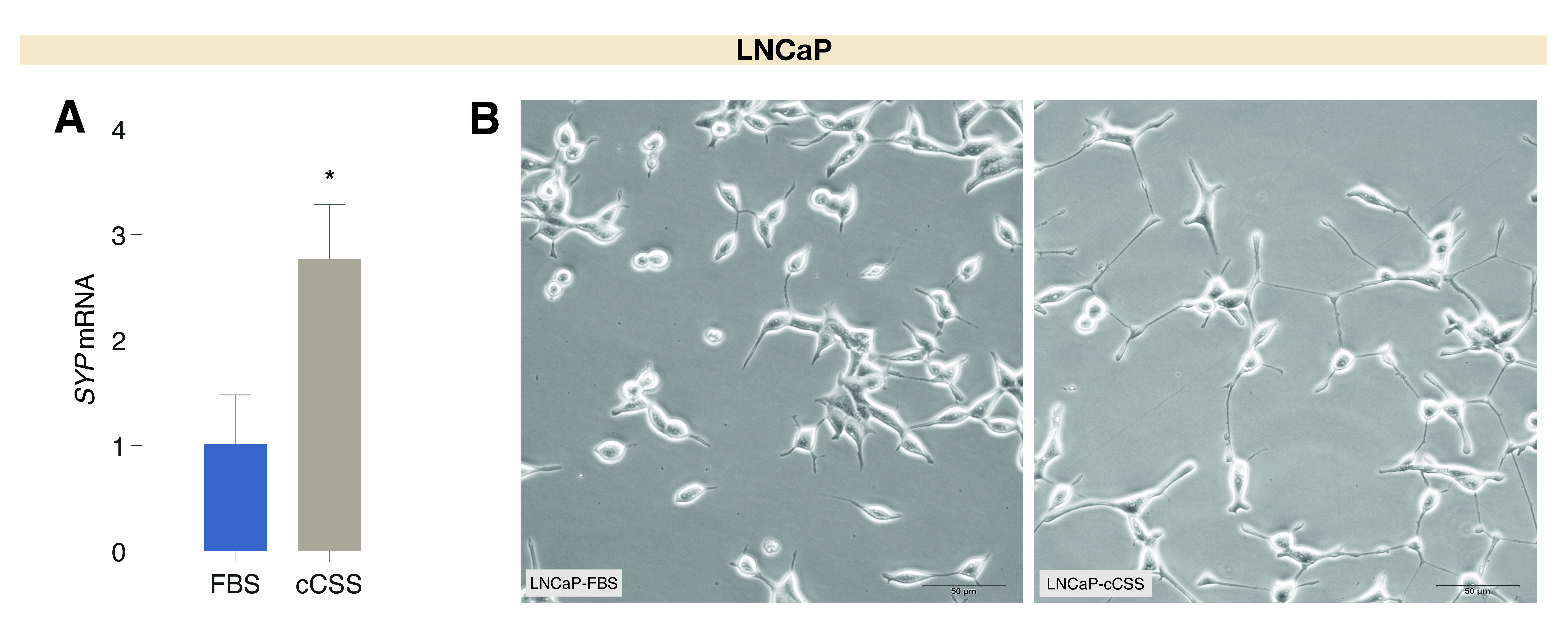

Supplement: S1 Fig — (A) LNCaP cells were maintained in complete growth media (FBS) and charcoal steroid-stripped (CSS) media for 10 mo (chronic CSS; cCSS). Chronic steroid starvation increased SYP mRNA expression. (B) Representative images (200X magnification) of LNCaP-FBS and LNCaP-cCSS at 90 days of cell culture maintenance are shown. The LNCaP-cCSS cells developed long cytoplasmic extensions resembling a neuron-like morphology. Bars represent mean ± SEM with statistical significance indicated by asterisks in Student’s t-test (*P < 0.05). (TIF) [file pone.0300413.s001.tif]

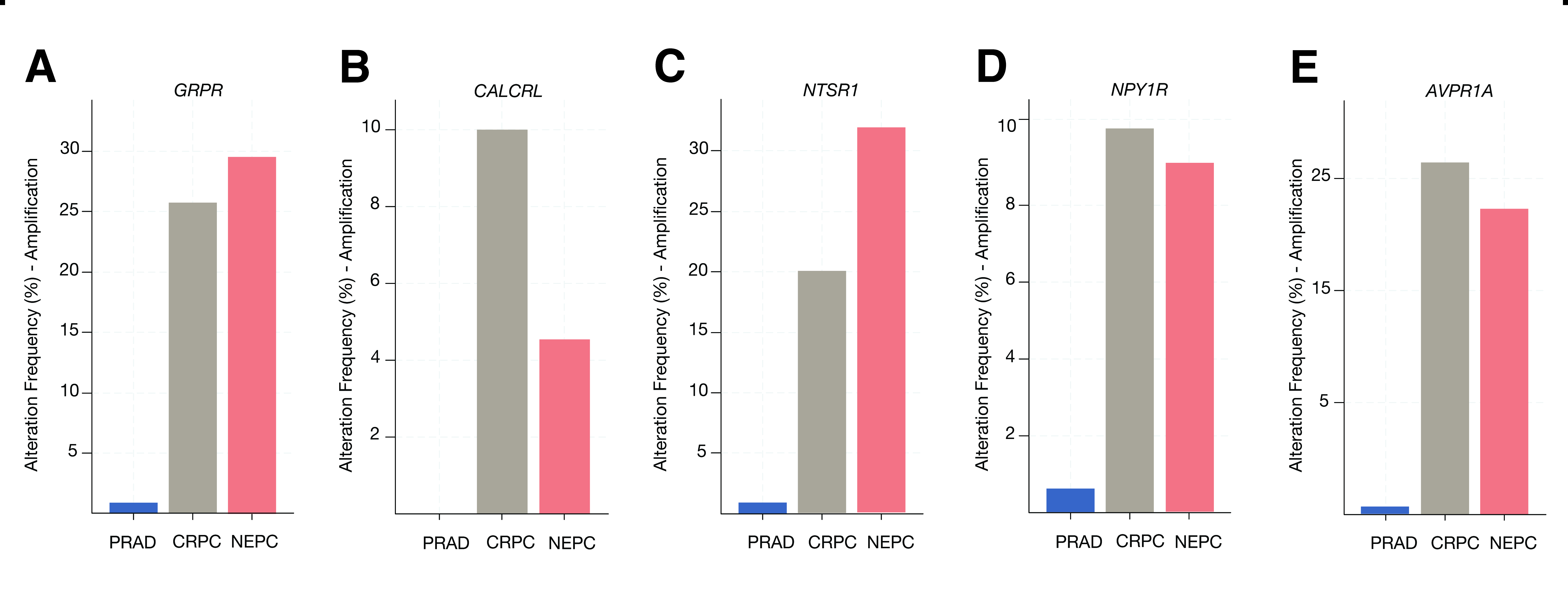

Supplement: S2 Fig — Gene amplification frequency of the neuropeptide receptor genes: (A) gastrin releasing peptide receptor (GRPR), (B) calcitonin receptor like receptor (CALCRL), (C) neurotensin receptor 1 (NTSR1), (D) neuropeptide Y receptor (NPY1R), (E) vasopressin receptor 1A (AVPR1A) were examined in the different types of PCa using publicly available RNA-seq and microarray copy number data [25], and visualized using the online visualization tool cBioPortal [27,34]. All five genes examined have higher amplification frequencies in the more advanced CRPC and NEPC stages relative to early-stage PRAD. (TIF) [file pone.0300413.s002.tif]

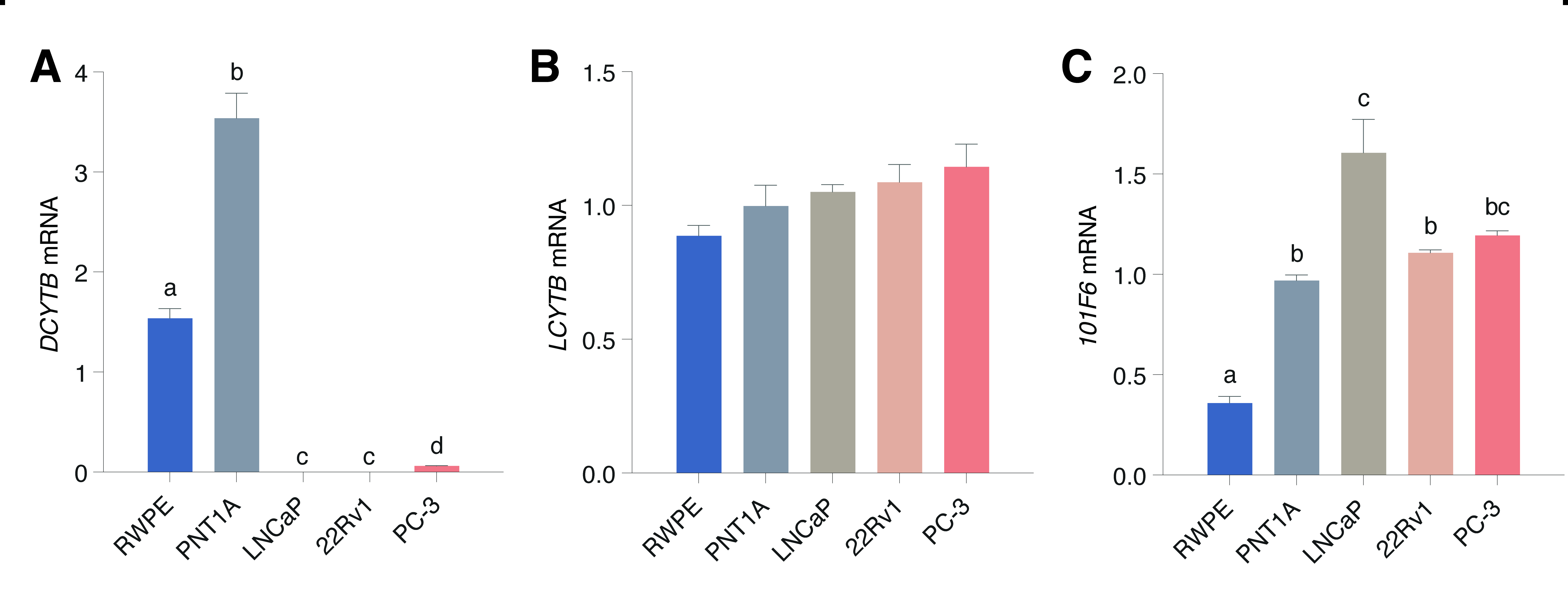

Supplement: S3 Fig — (A) The duodenal CYB561 homolog, DCYTB, shows high expression levels in the normal cell lines RWPE-1 and PNT1A. (B) The expression of lysosomal homolog, LCYTB, is similar across all cell lines analyzed. (C) The annotated tumor suppressor 101F6 exhibits high expression in LNCaP cells. Bars represent mean ± SEM with statistically significant differences determined through one-way ANOVA followed by Tukey’s post-hoc test (P < 0.05; means with the same letter are not statistically different). (TIF) [file pone.0300413.s003.tif]

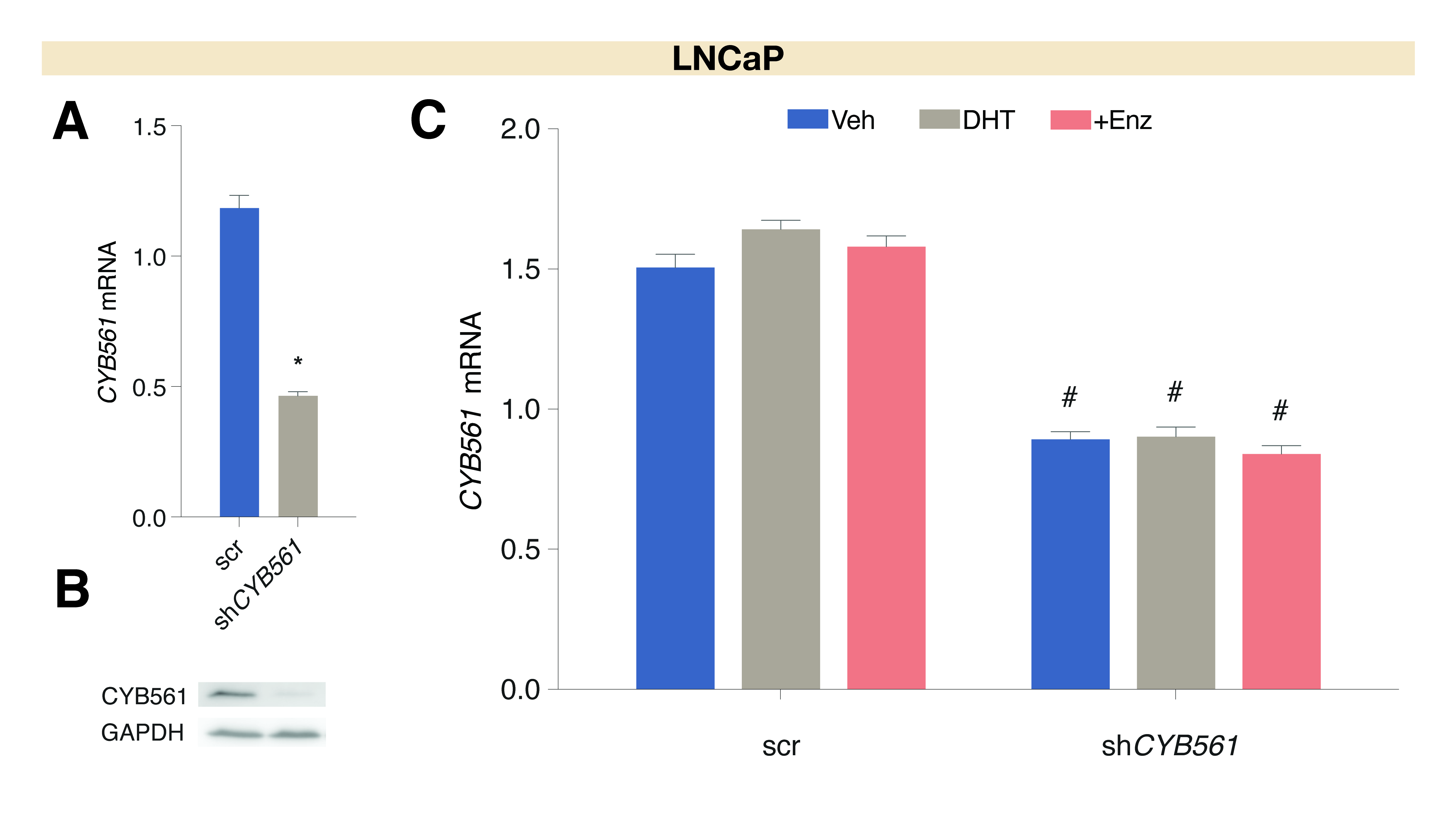

Supplement: S5 Fig — (A) Effective CYB561 knockdown was achieved at the (A) mRNA level by 74.24% as determined by RT-qPCR that was reflected at (B) the protein level as determined by western blot analysis in LNCaP cells transduced with shCYB561 compared to the scrambled (scr) shRNA control. (C) Hormone treatment did not alter CYB561 expression in scr shRNA and shCYB561 cells. Bars represent mean ± SEM with statistically significant differences determined through Student’s t-test for the effect of CYB561 knockdown (*P < 0.0001) or the effect of CYB561 knockdown between the same hormone treatment (#P < 0.0001). (TIF) [file pone.0300413.s005.tif]

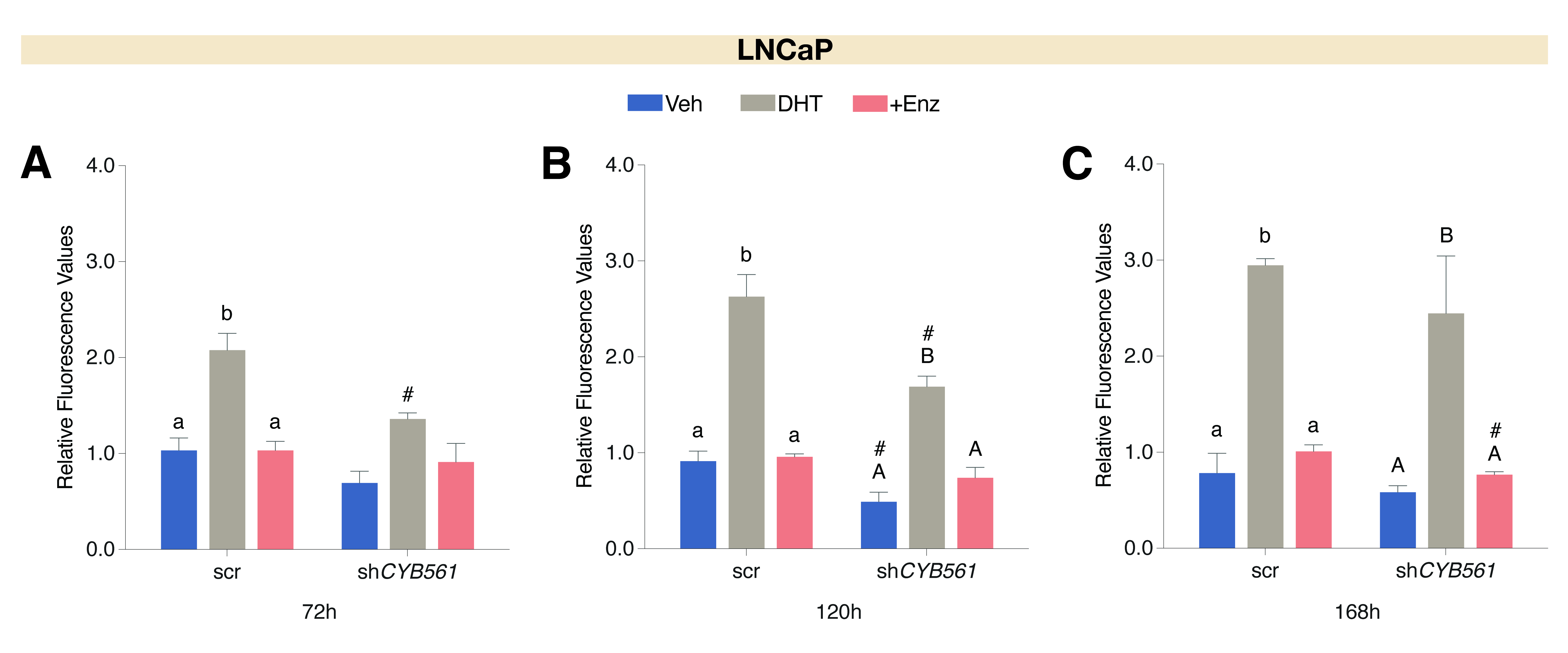

Supplement: S6 Fig — (A-C) Proliferation rates of transduced LNCaP cells upon hormone treatment were measured every 72 hr over the course of one week. Knockdown of CYB561 reduced the proliferative effects of DHT at (A) 72 hr and (B) 120 hr post-hormone treatment. Interestingly, CYB561 knockdown enhanced the repressive effect of Enz on cell proliferation at the (C) 168 hr timepoint. Data points and bars represent mean ± SEM with statistically significant differences determined through one-way ANOVA followed by Tukey’s post-hoc test for effect of hormone treatment (P < 0.05; means with the same letter are not statistically different) and Student’s t-test for the effect of CYB561 knockdown between the same hormone treatment (#P < 0.01). (TIF) [file pone.0300413.s006.tif]

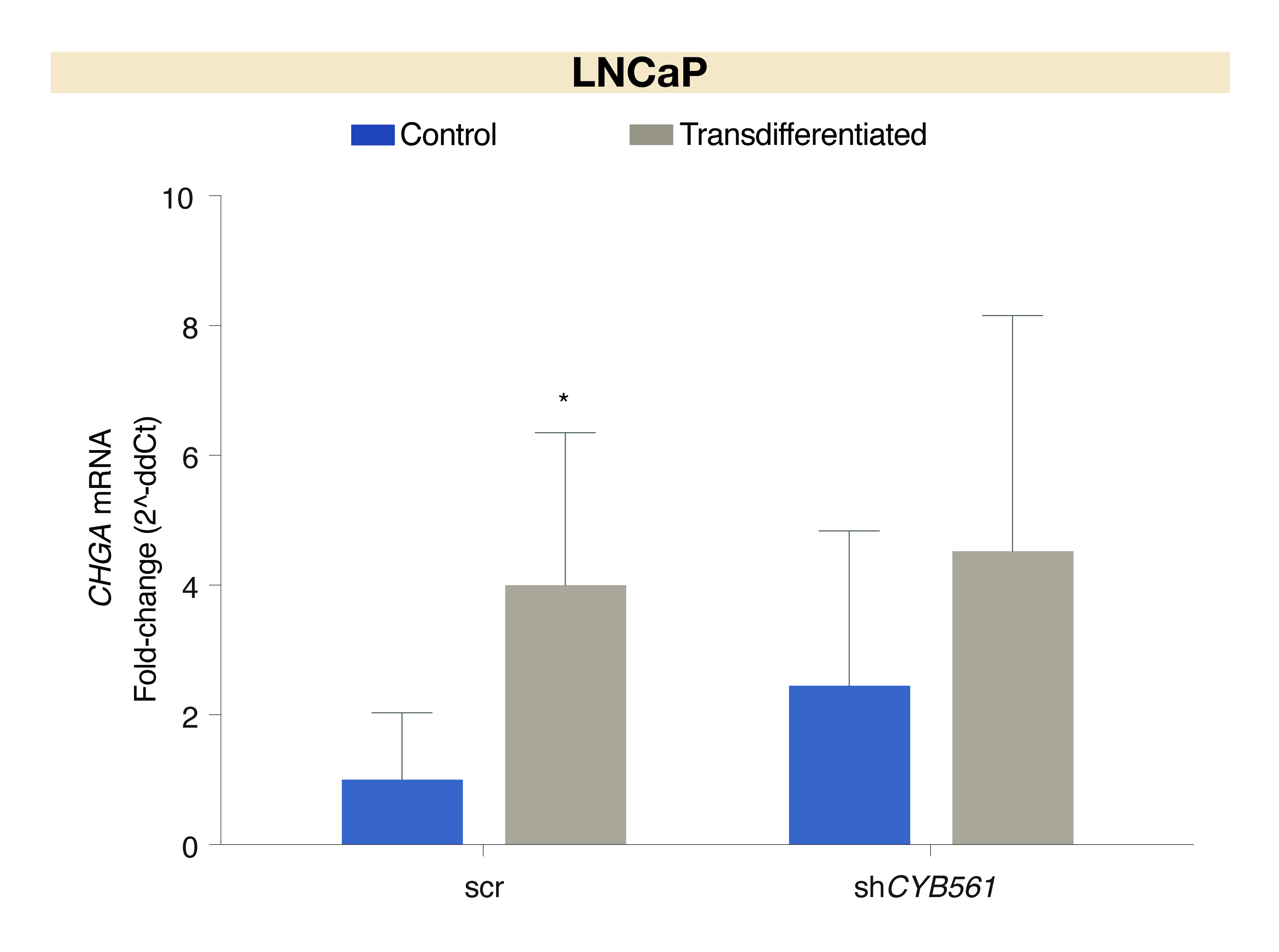

Supplement: S7 Fig — LNCaP cells transduced with the scrambled (scr) shRNA control and shCYB561 were grown and maintained in complete media (control) or transdifferentiation media for 14 days. Transdifferentiation induced a slight increase in relative CHGA mRNA levels in scr control cells (two-way ANOVA; Treatment factor: P = 0.0315; Knockdown factor: P = 0.0714). Bars represent fold-change values ± SEM of ΔCt with statistically significant differences determined through two-way ANOVA for main effects of treatment and CYB561 knockdown and Student’s t-test for the individual effects of treatment within an shRNA type (*P < 0.01). (TIF) [file pone.0300413.s007.tif]

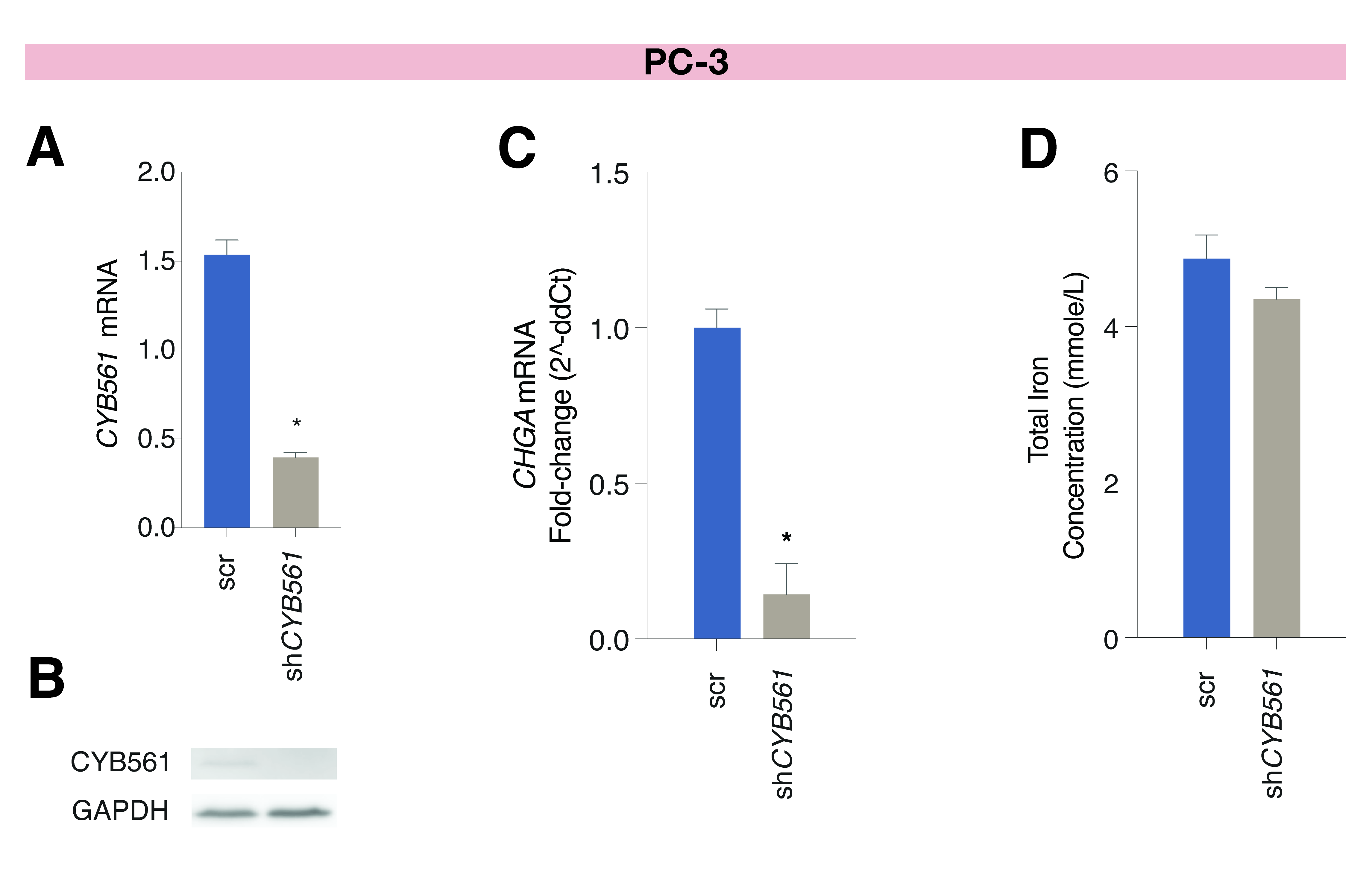

Supplement: S9 Fig — (A-B) Validation of CYB561 knockdown in PC-3 cells showed that (A) CYB561 mRNA expression was reduced by 74.23% at the mRNA level which was (B) reflected at the level of protein expression in PC-3 cells transduced with shCYB561 compared to the scrambled (scr) shRNA control. Knockdown of CYB561 (C) decreased CHGA mRNA expression but (D) did not affect total iron concentration in PC-3 cells. Bars represent mean ± SEM for CYB561 mRNA and total iron concentration plots while bars represent relative fold-change values ± SEM of the computed ΔCt values for CHGA plots. Statistically significant differences due to CYB561 knockdown were determined through Student’s t-test (*P < 0.0001). (TIF) [file pone.0300413.s009.tif]
