# Supplementary material for: CYB561 supports the neuroendocrine phenotype in castration-resistant prostate cancer
Source: PLoS One. 2024 May 13;19(5):e0300413. doi: 10.1371/journal.pone.0300413 (PMC11090301; doi:10.1371/journal.pone.0300413)
Supplement: S1 Table — (DOCX) [file pone.0300413.s010.docx]

**Supplementary Table**

**CYB561 supports the neuroendocrine phenotype in castration-resistant prostate cancer**

Romie Angelo G. Azur^¶^, Kevin Christian V. Olarte^¶^, Weand S. Ybañez, Alessandria Maeve M. Ocampo and Pia D. Bagamasbad^*^

National Institute of Molecular Biology and Biotechnology, University of the Philippines Diliman, Quezon City, Philippines

^*^ Corresponding author:

Email: pdbagamasbad@up.edu.ph

^¶^These authors contributed equally to this work.

**S1 Table. Primers used to generate the pLKO.1-sh*CYB561* construct for lentiviral transduction.**

| h*CYB561-1* shRNA | Forward | CCGGGAGTCCCTCCAGCCTGAATAACTCGAGTTATTCAGGCTGGAGGGACTCTTTTTG |
| --- | --- | --- |
|  | Reverse | AATTCAAAAAGAGTCCCTCCAGCCTGAATAACTCGAGTTATTCAGGCTGGAGGGACTC |
| h*CYB561-3* shRNA | Forward | CCGGGCACATCTTTGCGCTCGTCATCTCGAGATGACGAGCGCAAAGATGTGCTTTTTG |
|  | Reverse | AATTCAAAAAGCACATCTTTGCGCTCGTCATCTCGAGATGACGAGCGCAAAGATGTGC |
