# Supplementary material for: CYB561 supports the neuroendocrine phenotype in castration-resistant prostate cancer
Source: PLoS One. 2024 May 13;19(5):e0300413. doi: 10.1371/journal.pone.0300413 (PMC11090301; doi:10.1371/journal.pone.0300413)
Supplement: S1 Raw images — (PDF) [file pone.0300413.s011.pdf]

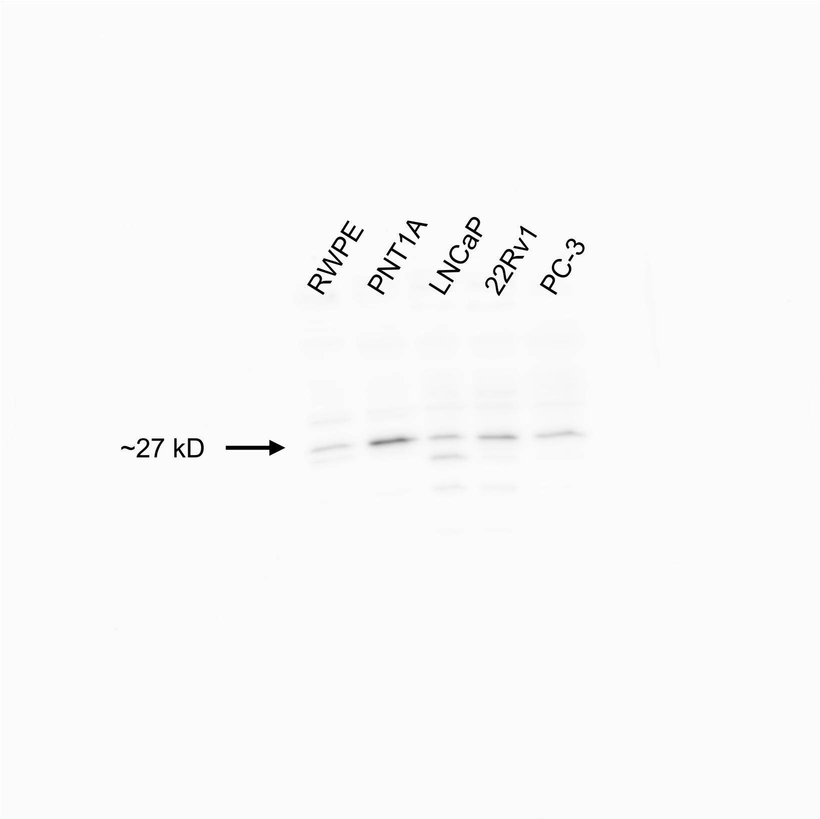

Image 1. Raw immunoblot image for CYB561 across five cell lines. Blot was used in Figure 1G.

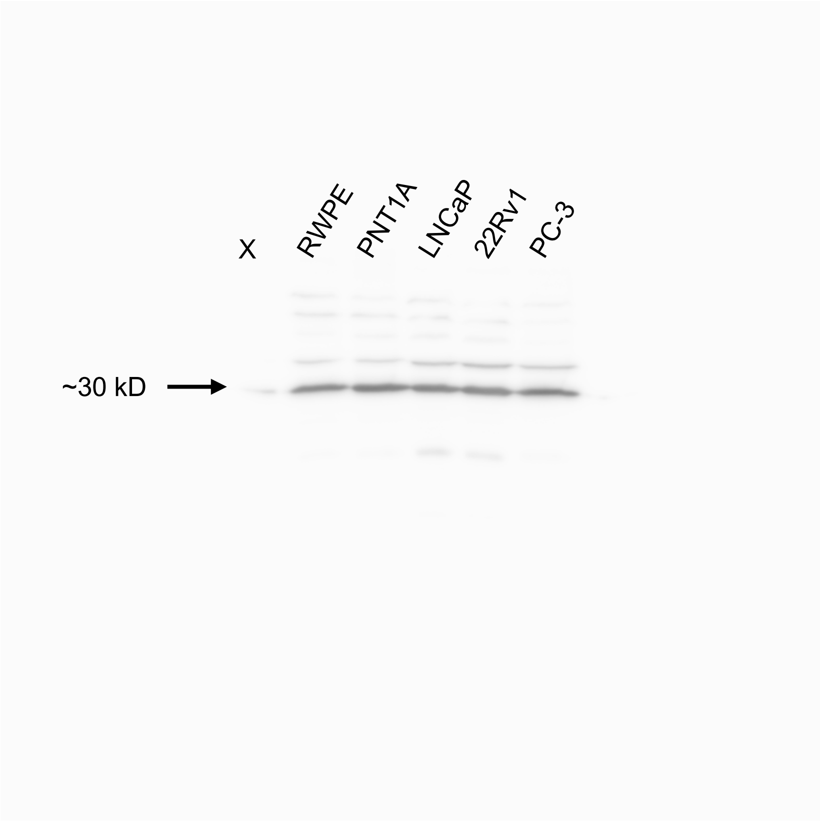

Image 2. Raw immunoblot image for the housekeeping protein GAPDH across five cell lines. The same blot for CYB561 was used to obtain this image. Blot was used in Figure 1G.

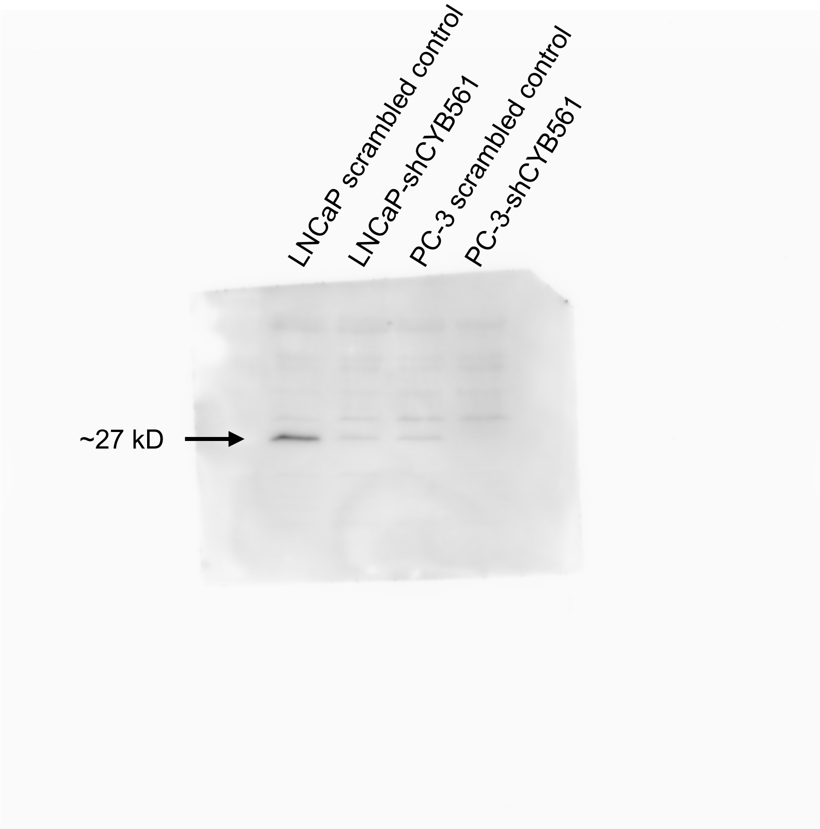

Image 3. Raw immunoblot image for CYB561 across two knockdown cell lines with their corresponding scrambled control. Blot was used in S5 Figure (Lane 1 and 2) and S9 Figure (Lane 3 and 4).

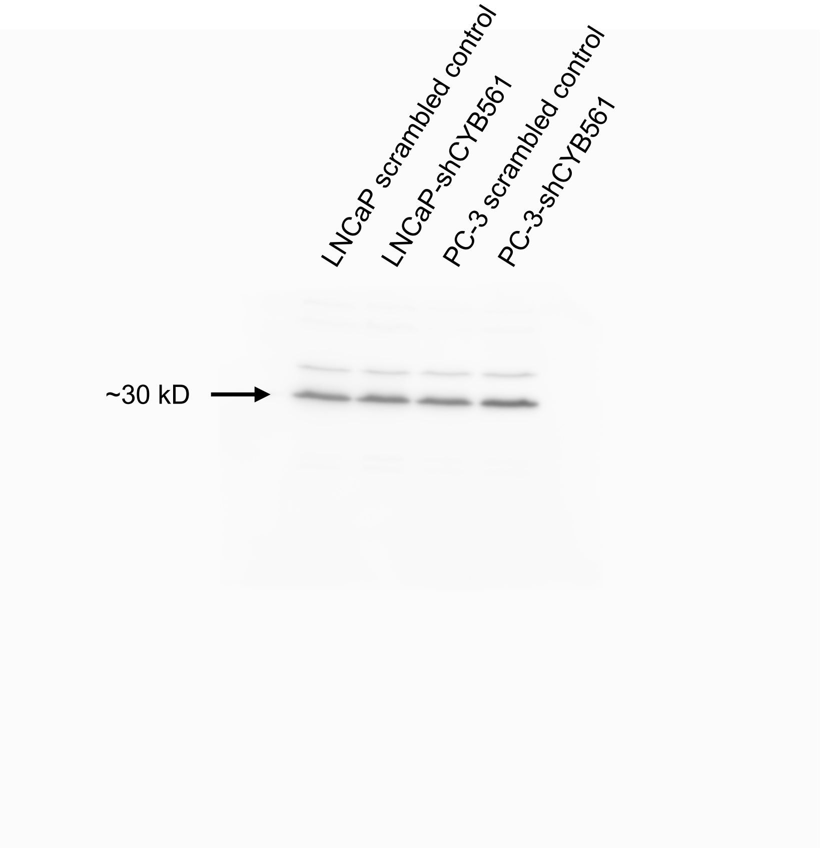

Image 4. Raw immunoblot image for the housekeeping protein GAPDH across two knockdown cell lines with their corresponding scrambled control. The same blot (Image 3) for CYB561 was used to obtain this image. Blot was used in S5 Figure (Lane 1 and 2) and S9 Figure (Lane 3 and 4).
