# Supplementary material for: CYB561 supports the neuroendocrine phenotype in castration-resistant prostate cancer
Source: PLoS One. 2024 May 13;19(5):e0300413. doi: 10.1371/journal.pone.0300413 (PMC11090301; doi:10.1371/journal.pone.0300413)
Supplement: Attachment — Submitted filename: [FINAL]_Response to reviewers_PLOS_CYB561 in PCA_02.23.2024.pdf [file pone.0300413.s012.pdf]

26 February 2024

Dear PLOS One Editorial Team:

We would like to express our gratitude for giving us the opportunity to resubmit a revised version of our manuscript, "CYB561 supports the neuroendocrine phenotype in castration-resistant prostate cancer". **We first submitted a version of this article to PLOS One (PONE-D-21-35614) in November 2021.** We appreciate the time and effort of the editorial team to help improve our work. The insightful feedback and comments led to major modifications that we believe have led to significant improvements to the manuscript. **In this current version of manuscript, the text in red incorporates all of the suggestions and edits recommended by the reviewers.** We hope that these changes are enough to satisfy your reservations regarding our previous submission. Below is a summary of revisions we have made and a point-by-point response to each of the comments of the Editor and Reviewer.

Editor's General Comments:

The emergence of NEPC is a major problem in managing the prostate cancer. The study seems interesting. However, the conclusions drawn are not consistent with the findings of the study. The discussion of the results is insufficient. The conclusions are drawn on the basis of data which is mRNA expression of CYB561 and genes (which are markers of NEPC).

1. **The knockdown studies show the association of genes with CBY561, however does not provide the functional role (as claimed to be dual by the authors), of protein.**
   We performed a western blot against CYB561 to confirm that it is expressed at the protein level in all cell lines we have used in this study (Fig 1G). We have also confirmed that *CYB561* mRNA knockdown is reflected at the level of protein expression in both LNCaP (SFig5B) and PC-3 cells (SFig9B). Please also refer to our response in no.3 below for supporting evidence of the effect of CYB561 depletion associated with the transdifferentiation-induced changes in the expression of neuroendocrine (NE) markers, and in cellular iron levels and iron response gene (IRG) expression.

   Expression of CYB561 protein has been added in the following:
   i. Methods section, lines 244-259:

   "Western Blot

   Model cell lines were seeded at a density of 2.0 x 10^6 cells per 100-mm culture dish and cultured until they reached 90% confluency. Total protein was extracted using RIPA buffer mixed with protease inhibitor cocktail (Millipore Merck, 539134) and quantified using Qubit® Protein Assay Kit (Thermo Fisher, Q33211). Proteins were separated by size by running 20 µg of the protein extract in 8% SDS-PAGE and transferred to Immuno-Blot PVDF Membrane (Bio-Rad, 1620174) using the Trans-Blot® SD Semi-Dry Transfer Cell (Bio-Rad) protocol. Non-specific interactions were blocked by incubating the membrane in TBS-T with 5% non-fat milk overnight at 4°C with constant agitation. To detect human CYB561 protein, an anti-CYB561 rabbit polyclonal antibody (Sigma-Aldrich, HPA014753) was used as the primary antibody and a goat polyclonal

anti-rabbit IgG conjugated to horseradish peroxidase (Millipore, 12-348) for the secondary antibody. To visualize protein bands, the ECL™ Prime Western Blotting Reagent (Cytiva, RPN2236) was used and images captured using the iBright CL100 Imaging System (Invitrogen). A housekeeping protein was also detected using the same blot by mild stripping and re-probing the membrane with an anti-GAPDH rabbit polyclonal antibody (Sigma-Aldrich, G9545-200).

ii. Results section

Lines 328-331:

"Lastly, western blot for CYB561 across the five cell lines showed higher CYB561 expression in 22rv1 and PC-3 cells compared to LNCaP cells, with the highest CYB561 protein expression in PNT1A suggesting that post-transcriptional regulation may play a role in modulating CYB561 protein expression (Fig 1G)."

Lines 351-354:

"Lentiviral-mediated transduction of sh*CYB561* in LNCaP cells effectively reduced *CYB561* mRNA expression by 74.24% relative to scrambled shRNA control (S5A Fig) which was also reflected at the protein level (S5B Fig), and remained unaltered with hormone treatment (S5C Fig)."

Lines 468-470:

"Lentiviral-mediated transduction of sh*CYB561* in PC-3 cells successfully reduced *CYB561* mRNA expression by 74.23% relative to scrambled shRNA control (S9A Fig) which was reflected at the level of protein expression (S9B Fig)."

We have also included the following to the Discussion section to expand the discussion of our results:

i. Role of neuroendocrine markers through PCa progression, lines 575-581:

"We also observed *PAM* expression to be downregulated by androgen treatment and upregulated by acute and chronic CSS conditions in LNCaP cells. Notably, when we did the same treatment in 22Rv1 cells, a PCa cell line known to be less reliant on androgen for growth and representative of the progression from PRAD to NEPC, the androgen-dependent downregulation of *PAM* was abolished (17), consistent with our hypothesis that PAM may be negatively regulated by androgen signaling. In the future, it would be interesting to look at the individual contribution of PAM to NEPC progression."

ii. Contribution of CYB561 ferreducatse activity in PCa progression, lines 585-597:

"While we cannot fully ascertain the relative levels of LIP without direct measurements of intracellular Fe^{2+} across cell lines, the pattern of IRG expression we observed points towards NEPC having higher LIP compared to PRAD. This is consistent with previous reports that showed metastatic PCa upregulating the autocrine hormone hepcidin to promote ferroportin degradation (43). This decrease in ferroportin expression effectively increases iron retention and promotes cancer cell survival (43). As for the significance of the ferrireductase function of CYB561 in PCa iron metabolism, knockdown of *CYB561* in LNCaP cells decreased Fe^{2+} concentration indicating that reduced CYB561 expression can lower cellular

ferrireductase activity. These results, along with the sensitizing effect of CYB561 knockdown to Enz, may indicate a potential contribution of CYB561 ferrireductase function towards supporting tumor growth and therapy resistance through increased cellular iron load (9). Furthermore, these findings point to the benefit of using ferrireductase inhibitors in combination with established anti-PCa drugs to delay or prevent castration resistance."

iii. Link between NE transdifferentiation and iron metabolism, lines 632-639:
"While a definitive link between iron and NE transdifferentiation is lacking, the observation that Fe^{2+} is required for the activity of the chromatin modifying enzyme JARID1B provides a possible mechanism on how intracellular iron levels influence the chromatin landscape and consequently contribute to NEPC reprogramming (47). JARID1B, a histone H3 lysine 4 demethylase upregulated in several cancer types including PCa, requires Fe^{2+} and α-ketoglutarate as cofactors for its demethylase activity (47). One can then infer that changes in the availability and metabolism of these factors can affect histone demethylase activity and consequently the chromatin landscape."

2. The association between proliferation and presence of CBY561 is interesting. There is major issue with hypothesis which states that CBY561 drives the progression of prostate cancer from adenocarcinoma to castration resistant thru NEPC phenotypes.

To more clearly state the rationale/hypothesis for this study and temper our conclusion to emphasize that CYB561 **contributes** towards PCa progression to NEPC, we have added the following in:

Introduction, lines 87-91:
"The elevated expression of *CYB561* in CRPC relative to metastatic PCa (11, 12), along with its dual role in neuropeptide signaling and iron metabolism, led us to explore a possible contextual relationship between CYB561, and transformation and maintenance of NEPC. Notably, the possible involvement of CYB561 in relation to CRPC development and NEPC has not been functionally established."

Introduction, lines 105-106:
"To our knowledge, this is the first study to establish the functional involvement of CYB561 in CRPC development and NEPC."

3. Authors used LNCaP, LNCaP-under css, and PC3 as models, and results vary. Whereas it is true that long-term treatment of LNCaP in CSS media causes the emergence of EMT, NEPC features, in LNCaP, the current study data does not show much change.
We respectfully disagree with this comment as we have clearly shown several lines of evidence that acute CSS media treatment of LNCaP cells transduced with scrambled shRNA control led to an increase in expression of NE markers (Fig 3B-D, SFig4C-F), increased Fe^{2+} concentration (Fig 4B and SFig8D), and altered the expression of IRGs (Fig 4C-E and SFig8E-F). These results are consistent with the known increase in NE marker expression upon

transdifferentiation to a NE phenotype and the iron-seeking behavior through cancer progression. More importantly, in the same figures cited, **we have shown that knocking down CYB561 expression was sufficient to significantly dampen the acute CSS-induced increase in NE marker expression and Fe^{2+} concentration, and alter the expression of IRG expression relative to the scrambled shRNA control.** Furthermore, we also provide evidence that a similar pattern can be observed upon chronic CSS treatment of LNCaP cells (SFig1, SFig4D-F, and SFig8E-F).

4. Intriguingly, knockdown of CBY561 reduced the growth of LNCaP cells suggesting the dependence of hormone-dependent prostate tumor cell viability on CBY561. Furthermore, the data where DHT treatment induced the mRNA expression of CBY561 and ENZ partially decreasing the expression suggests CBY561 could be partially regulated by androgen pathway. Nevertheless, PC3 expresses CBY561.This does not provide evidence about its role in progression.

   We respectfully disagree with this comment as we have shown that DHT and Enz treatment of LNCaP (Fig2A) and 22rv1 cells (Fig 2D) did not significantly change the levels of *CYB561* mRNA expression. In LNCaP cells, we also show that treatment with acute CSS (SFig4B) or chronic CSS (SFig4E) media did not significantly alter *CYB561* expression, further strengthening our conclusion that *CYB561* expression is not dependent on androgen regulation.

Editor's Specific Comments:

1. Authors should test hypothesis in established cell models such as C42B, 22Rv1 (under long-term and short-term treatments of ENZ or bicalutamide). This is because emergence of NEPC is observed after treatment in clinic.

We have added 22rv1 as another model to serve as a midpoint between the androgen-responsive PRAD-model LNCaP and the androgen-indolent NEPC-model PC-3. Amongst the landmark features of 22rv1 is its expression of KLK3 and AR despite its weak response to androgen stimulation (1). We showed that *CYB561* mRNA and protein levels in 22rv1 cells are higher or comparable to LNCaP (*CYB561* mRNA: Fig 1E; CYB561 protein: Fig 1G) while baseline *PAM* expression in 22rv1 is higher when compared to LNCaP and lower relative to PC-3 (Fig 1E-F). We also looked at the effect of short-term hormone stimulation in 22rv1 where we find that both *CYB561* and *PAM* levels did not change upon androgen-stimulation (Fig 2C-D). This contrasts what we observed in LNCaP (Fig2A-B) where *PAM* expression decreases upon treatment with DHT which further supports the idea that it may be *PAM* that is negatively regulated by androgens. While we agree with the reviewer that NEPC emergence is usually seen after ADT, we decided not to pursue long-term treatment of 22rv1 with enzalutamide or bicalutamide since 22rv1 was serially propagated in castrated-resistant mice, its molecular features should be similar to prostate adenocarcinoma cells subjected to long-term treatment with AR antagonists (1). Below are revisions and additional experiments performed related to 22rv1 cells.

i. Description of 22rv1 cells in Methods section:

Lines 116-120:
"The 22Rv1 cell line, a model of the progression of androgen-dependent prostate adenocarcinoma (PRAD) to NEPC, is a xenograft-derivative of CWR22 PRAD cells after serial propagation in castrated mice (17). Like LNCaP, 22Rv1 cells also express AR and PSA but has weak response to dihydrotestosterone (DHT) and androgen analogues."

Lines 123-125:
"PNT1A (Cat. No. 95012614) and LNCaP (Cat. No. 89110211) cells were purchased from the European Collection of Authenticated Cell Cultures, while 22Rv1 cells were obtained from the American Type Culture Collection (Cat. No. CRL-2505)."

Lines 200-203:
"The same protocol was adapted to measure the effect of paracrine signaling on the proliferation rates of parental PNT1A (1.0×10^3 cells/well), LNCaP (3.0×10^3 cells/well), and 22rv1 (3.0×10^3 cells/well) cells treated with conditioned media."

ii. We measured the baseline expression of CYB561 mRNA (Fig 1E) and protein (Fig 1G) in all cell lines, including 22rv1. We included 22rv1 in our analysis of the mRNA expression of *CYB561* homologues (SFig3A-C) and IRG mRNA levels (SFig8A-C) all cell lines used in the study. We also used 22rv1 cells to determine the effect of DHT and Enz treatment on *CYB561* expression. These revisions are incorporated in the manuscript as follows:

<u>Baseline expression of CYB561 to include 22rv1 cells</u>. Results, lines 321-324:
"To support our *in silico* findings, we measured the baseline expression levels of *CYB561* mRNA in prostate cell lines, and found that *CYB561* is elevated in metastatic LNCaP and 22Rv1 cells, and in the NEPC cell model PC-3 relative to normal prostate epithelial cell lines RWPE and PNT1A (Fig 1E)."

<u>Baseline expression of CYB561 to include 22rv1 cells</u> Results, lines 328-331:
"Lastly, western blot for CYB561 across the five cell lines showed higher CYB561 expression in 22rv1 and PC-3 cells compared to LNCaP cells, with the highest CYB561 protein expression in PNT1A suggesting that post-transcriptional regulation may play a role in modulating CYB561 protein expression (Fig 1G)."

<u>Baseline expression of CYB561 to include 22rv1 cells</u>. Results, lines 418-421:
Notably, LNCaP and 22Rv1 cells show a similar pattern of IRG expression except for the higher *FPN1* mRNA in 22rv1. PC-3 cells consistently showed highest basal expression of *TFRC* and *FTH* across all cell prostate cell lines.

<u>Baseline expression of CYB561 to include 22rv1 cells</u>. Discussion, lines 654-657:
"The comparative pattern of IRG expression across the different PCa cell lines are consistent with a consequent increase of LIP levels in PC-3 in comparison to LNCaP and 22Rv1 cells."

Effect of DHT and Enz treatment on *CYB561* expression in 22rv1 cells. Results, lines 339-341:
"On the other hand, both *CYB561* (Fig 2C) and *PAM* mRNA levels (Fig 2D) did not change in response to DHT or Enz treatment in 22Rv1 cells."

Effect of DHT and Enz treatment on *CYB561* expression in 22rv1 cells. Results, lines 346-347:
"However, this androgen-dependent regulation of *PAM* is no longer observed in 22Rv1 cells."

Effect of DHT and Enz treatment on *CYB561* expression in 22rv1 cells. Discussion section, lines 575-581:
"We also observed *PAM* expression to be downregulated by androgen treatment and upregulated by acute and chronic CSS conditions in LNCaP cells. Notably, when we did the same treatment in 22Rv1 cells, a PCa cell line known to be less reliant on androgen for growth and representative of the progression from PRAD to NEPC, the androgen-dependent downregulation of *PAM* was abolished (17), consistent with our hypothesis that PAM may be negatively regulated by androgen signaling. In the future, it would be interesting to look at the individual contribution of PAM to NEPC progression."

iii. We measured the cell viability of 22rv1 cells upon growth in PC3-conditioned media (Fig 5H-I) where we observed that conditioned media from PC3-sh*CYB561* cells did not support cell proliferation of 22rv1 cells relative to conditioned media collected from PC3-scrambled shRNA cells. This provides additional supporting evidence that neuroendocrine cell-derived neuropeptides, such as those produced by NEPC cells, may be essential in supporting the viability of surrounding PRAD cells in a tumor microenvironment.

Results, lines 476-482:
"We used conditioned media derived from PC-3 scrambled shRNA control or sh*CYB561* cell lines as growth media for PNT1A prostate cells, LNCaP and 22Rv1 PCa cells. Treatment with conditioned media from PC-3 scrambled shRNA control and the positive control media were able to maintain cell survival and proliferation while treatment with PC-3 sh*CYB561*-derived conditioned media and negative control media led to a significant decline in the survival of cells across all three cell lines (Figs 5D-I)."

2. Authors should show the effect of treatments on protein level, cytoplasmic and nuclear AR, and establish correlation with CBY561 protein.
We have already provided evidence on the CYB561 protein expression as detailed in Editor's General Comment #1.

While we agree about the significant role of AR signaling in PRAD and the transition to NEPC, several papers have already shown that growing LNCaP in serum-starved media reduces AR protein levels considerably (2, 3, 4), and that this is accompanied by extensive nuclear AR localization (3) which is in line with the observation that ADT provides selective pressure on PRAD to reduce its dependence on androgens to induce the AR signaling pathway (5). More

importantly, AR localization is not a definitive indicator of AR activity. In LNCaP cells, we have shown the effect of our treatment on AR activity as shown by the expected changes in *KLK3* (PSA) expression upon DHT and Enz treatment (Fig2D), acute CSS media treatment (SFig4A), and chronic CSS media treatment (SFig4D). Therefore, the effect of various treatments on *KLK3* expression as we have shown is a better indicator of AR activity.

3. Authors shall provide data of CBY561 protein vis-à-vis NEPC markers in cells at different stages of phenotype change, morphology change, and pH change during serum starvation. The confocal microscopy-based protein changes and western blot would suffice for the evidence. These data should be compared with established AR-positive CRPC models.

Similar to Editor's Specific Comment #2, a previous study has already described the phenotypic and morphological changes that accompany the transition from PRAD to NEPC upon serum starvation, specifically that the cells transitioned towards a more circular morphology, a distinctly distended nuclei, and the formation of clusters and foci (3). Moreover, the proliferation rate of the reprogrammed cells had become slower compared to their control counterparts. In agreement to this, the reported phenotypic changes were also observed in our transdifferentiation experiments.

Reviewer's Comments:

1. Authors should add the C42b cell line for their study. This cell line is considered as one of the best AR positive CRPC models. If they observe the same results with C42b, the impact of the paper will increase a lot.

   Please see response to Editor's Specific comment #1.

On behalf of my co-authors, we hope that revisions and clarifications we have made are sufficient to merit acceptance of our manuscript.

Sincerely,

Pia D. Bagamasbad, Ph.D.
Professor
National Institute of Molecular Biology and Biotechnology
University of the Philippines
Diliman, Quezon City, Philippines, 1101
Email: pdbagamasbad@up.edu.ph

Literature Cited

1. Sramkoski RM, Pretlow TG, 2nd, Giaconia JM, Pretlow TP, Schwartz S, Sy MS, et al. A new human prostate carcinoma cell line, 22Rv1. In Vitro Cell Dev Biol Anim. 1999;35(7):403-9.
2. Ciarlo M, Benelli R, Barbieri O, Minghelli S, Barboro P, Balbi C, et al. Regulation of neuroendocrine differentiation by AKT/hnRNPK/AR/beta-catenin signaling in prostate cancer cells. Int J Cancer. 2012;131(3):582-90.
3. Nouri M, Caradec J, Lubik AA, Li N, Hollier BG, Takhar M, et al. Therapy-induced developmental reprogramming of prostate cancer cells and acquired therapy resistance. Oncotarget. 2017;8(12):18949-67.
4. Yuan T, Veeramani, S., Lin, F., Kondrikou, D., Zelivianski, S., Igawa, T., Karan, D., Batra, S., & Lin, M. Androgen deprivation induces human prostate epithelial neuroendocrine differentiation of androgen-sensitive LNCaP cells. Endocrine Related Cancer. 2006;13(1):151-67.
5. Karantanos T, Corn PG, Thompson TC. Prostate cancer progression after androgen deprivation therapy: mechanisms of castrate resistance and novel therapeutic approaches. Oncogene. 2013;32(49):5501-11.

Date: Jan 05 2022 10:28PM
To: "Pia Dano Bagamasbad" piab@mbb.upd.edu.ph;piab@umich.edu
From: "PLOS ONE" plosone@plos.org
Subject: PLOS ONE Decision: Revision required [PONE-D-21-35614]

PONE-D-21-35614
Dual role of CYB561 supports the neuroendocrine phenotype in castration-resistant prostate cancer
PLOS ONE

Dear Dr. Bagamasbad,

Thank you for submitting your manuscript to PLOS ONE. After careful consideration, we feel that it has merit but does not fully meet PLOS ONE's publication criteria as it currently stands. Therefore, we invite you to submit a revised version of the manuscript that addresses the points raised during the review process.

Please submit your revised manuscript by Feb 19 2022 11:59PM. If you will need more time than this to complete your revisions, please reply to this message or contact the journal office at plosone@plos.org. When you're ready to submit your revision, log on to https://www.editorialmanager.com/pone/ and select the 'Submissions Needing Revision' folder to locate your manuscript file.

Please include the following items when submitting your revised manuscript:

- A rebuttal letter that responds to each point raised by the academic editor and reviewer(s). You should upload this letter as a separate file labeled 'Response to Reviewers'.
- A marked-up copy of your manuscript that highlights changes made to the original version. You should upload this as a separate file labeled 'Revised Manuscript with Track Changes'.
- An unmarked version of your revised paper without tracked changes. You should upload this as a separate file labeled 'Manuscript'.

If you would like to make changes to your financial disclosure, please include your updated statement in your cover letter. Guidelines for resubmitting your figure files are available below the reviewer comments at the end of this letter.

If applicable, we recommend that you deposit your laboratory protocols in protocols.io to enhance the reproducibility of your results. Protocols.io assigns your protocol its own identifier (DOI) so that it can be cited independently in the future. For instructions see: https://journals.plos.org/plosone/s/submission-guidelines#loc-laboratory-protocols. Additionally, PLOS ONE offers an option for publishing peer-reviewed Lab Protocol articles, which describe protocols hosted on protocols.io. Read more information on sharing protocols at https://plos.org/protocols?utm_medium=editorial-email&utm_source=authorletters&utm_campaign=protocols.

We look forward to receiving your revised manuscript.

Kind regards,

Mohammad Saleem
Academic Editor
PLOS ONE

Additional Editor Comments:

The emergence of NEPC is a major problem in managing the prostate cancer. The study seems interesting. However, the conclusions drawn are not consistent with the findings of the study. The discussion of the results is insufficient. The conclusions are drawn on the basis of data which is mRNA expression of CYB561 and genes (which are markers of NEPC). The knockdown studies show the association of genes with CBY561, however does not provide the functional role (as claimed to be dual by the authors), of protein. The association between proliferation and presence of CBY561 is interesting. There is major issue with hypothesis which states that CBY561 drives the progression of prostate cancer from adenocarcinoma to castration resistant thru NEPC phenotypes. Authors used LNCaP, LNCaP-under css, and PC3 as models, and results vary. Whereas it is true that long-term treatment of LNCaP in CSS media causes the emergence of EMT, NEPC features, in LNCaP,

the current study data does not show much change. Intriguingly, knockdown of CBY561 reduced the growth of LNCaP cells suggesting the dependence of hormone-dependent prostate tumor cell viability on CBY561. Furthermore, the data where DHT treatment induced the mRNA expression of CBY561 and ENZ partially decreasing the expression suggests CBY561 could be partially regulated by androgen pathway. Nevertheless, PC3 expresses CBY561.This does not provide evidence about its role in progression. Following data is required to consider the manuscript for further review:
1. Authors should test hypothesis in established cell models such as C42B, 22Rv1 (under long-term and short-term treatments of ENZ or bicalutamide). This is because emergence of NEPC is observed after treatment in clinic.
2. Authors should show the effect of treatments on protein level, cytoplasmic and nuclear AR, and establish correlation with CBY561 protein.
3. Authors shall provide data of CBY561 protein vis-à-vis NEPC markers in cells at different stages of phenotype change, morphology change, and pH change during serum starvation. The confocal microscopy-based protein changes and western blot would suffice for the evidence. These data should be compared with established AR-positive CRPC models.

Journal requirements:

When submitting your revision, we need you to address these additional requirements.

1. Please ensure that your manuscript meets PLOS ONE's style requirements, including those for file naming. The PLOS ONE style templates can be found at

https://journals.plos.org/plosone/s/file?id=wjVg/PLOSOne_formatting_sample_main_body.pdf and

https://journals.plos.org/plosone/s/file?id=ba62/PLOSOne_formatting_sample_title_authors_affiliations.pdf

2. In your Data Availability statement, you have not specified where the minimal data set underlying the results described in your manuscript can be found. PLOS defines a study's minimal data set as the underlying data used to reach the conclusions drawn in the manuscript and any additional data required to replicate the reported study findings in their entirety. All PLOS journals require that the minimal data set be made fully available. For more information about our data policy, please see http://journals.plos.org/plosone/s/data-availability.

Upon re-submitting your revised manuscript, please upload your study's minimal underlying data set as either Supporting Information files or to a stable, public repository and include the relevant URLs, DOIs, or accession numbers within your revised cover letter. For a list of acceptable repositories, please see http://journals.plos.org/plosone/s/data-availability#loc-recommended-repositories. Any potentially identifying patient information must be fully anonymized.

Important: If there are ethical or legal restrictions to sharing your data publicly, please explain these restrictions in detail. Please see our guidelines for more information on what we consider unacceptable restrictions to publicly sharing data: http://journals.plos.org/plosone/s/data-availability#loc-unacceptable-data-access-restrictions. Note that it is not acceptable for the authors to be the sole named individuals responsible for ensuring data access.

We will update your Data Availability statement to reflect the information you provide in your cover letter.

[Note: HTML markup is below. Please do not edit.]

Reviewers' comments:

Reviewer's Responses to Questions

Comments to the Author

1. Is the manuscript technically sound, and do the data support the conclusions?

The manuscript must describe a technically sound piece of scientific research with data that supports the conclusions. Experiments must have been conducted rigorously, with appropriate controls, replication, and sample sizes. The conclusions must be drawn appropriately based on the data presented.

Reviewer #1: Yes

---

2. Has the statistical analysis been performed appropriately and rigorously?

Reviewer #1: Yes

---

3. Have the authors made all data underlying the findings in their manuscript fully available?

The PLOS Data policy requires authors to make all data underlying the findings described in their manuscript fully available without restriction, with rare exception (please refer to the Data Availability Statement in the manuscript PDF file). The data should be provided as part of the manuscript or its supporting information, or deposited to a public repository. For example, in addition to summary statistics, the data points behind means, medians and variance measures should be available. If there are restrictions on publicly sharing data—e.g. participant privacy or use of data from a third party—those must be specified.

Reviewer #1: Yes

---

4. Is the manuscript presented in an intelligible fashion and written in standard English?

PLOS ONE does not copyedit accepted manuscripts, so the language in submitted articles must be clear, correct, and unambiguous. Any typographical or grammatical errors should be corrected at revision, so please note any specific errors here.

Reviewer #1: Yes

---

5. Review Comments to the Author

Please use the space provided to explain your answers to the questions above. You may also include additional comments for the author, including concerns about dual publication, research ethics, or publication ethics. (Please upload your review as an attachment if it exceeds 20,000 characters)

Reviewer #1: This is an interesting study. I have only one comment.
1. Authors should add the C42b cell line for their study. This cell line is considered as one of the best AR positive CRPC models. If they observe the same results with C42b, the impact of the paper will increase a lot.

---

6. PLOS authors have the option to publish the peer review history of their article (what does this mean?). If published, this will include your full peer review and any attached files.

If you choose “no”, your identity will remain anonymous but your review may still be made public.

Do you want your identity to be public for this peer review? For information about this choice, including consent withdrawal, please see our Privacy Policy.

Reviewer #1: No

[NOTE: If reviewer comments were submitted as an attachment file, they will be attached to this email and accessible via the submission site. Please log into your account, locate the manuscript record, and check for the action link "View Attachments". If this link does not appear, there are no attachment files.]

While revising your submission, please upload your figure files to the Preflight Analysis and Conversion Engine (PACE) digital diagnostic tool, https://pacev2.apexcovantage.com/. PACE helps ensure that figures meet PLOS requirements. To use PACE, you must first register as a user. Registration is free. Then, login and navigate to the UPLOAD tab, where you will find detailed instructions on how to use the tool. If you encounter any issues or have any questions when using PACE, please email PLOS at figures@plos.org. Please note that Supporting Information files do not need this step.

In compliance with data protection regulations, you may request that we remove your personal registration details at any time. (Remove my information/details). Please contact the publication office if you have any questions.
